# Metabolic phenotype in Darier disease: a cross-sectional clinical study

**DOI:** 10.1186/s13098-020-0520-0

**Published:** 2020-02-05

**Authors:** Tara Ahanian, Philip Curman, Ivone U. S. Leong, Kerstin Brismar, Etty Bachar-Wikstrom, Martin Cederlöf, Jakob D. Wikstrom

**Affiliations:** 1 Dermatology and Venereology Division, Department of Medicine (Solna), Karolinska Institutet, Stockholm, Sweden; 2 Dermato-Venereology Clinic, Karolinska University Hospital, Stockholm, Sweden; 3 The Rolf Luft Research Center for Diabetes and Endocrinology, Karolinska Institutet, Stockholm, Sweden; 4 Department of Medical Epidemiology and Biostatistics, Karolinska Institutet, Stockholm, Sweden

**Keywords:** Darier disease, Diabetes, Endoplasmic reticulum, Calcium, ER stress, Glucose, Insulin, Sarcoendoplasmic-reticulum ATPase 2 (SERCA2)

## Abstract

**Background:**

Human data supporting a role for endoplasmic reticulum (ER) stress and calcium dyshomeostasis in diabetes is scarce. Darier disease (DD) is a hereditary skin disease caused by mutations in the *ATP2A2* gene encoding the sarcoendoplasmic-reticulum ATPase 2 (SERCA2) calcium pump, which causes calcium dyshomeostasis and ER stress. We hypothesize that DD patients have a diabetes-like metabolic phenotype and the objective of this study was to examine the association between DD with impaired glucose tolerance and diabetes.

**Methods:**

Cross-sectional clinical study on 25 DD patients and 25 matched controls. Metabolic status was assessed primarily by fasting blood glucose, oral glucose tolerance test, HOMA2-%S (insulin resistence) and HOMA2-%B (beta cell function).

**Results:**

DD subjects showed normal oral glucose tolerance test and HOMA2-%S, while fasting blood glucose was lower and c-peptide as well as HOMA2-%B was higher.

**Conclusion:**

Increased HOMA2-%B values are indicative of increased basal insulin secretion which is a type of beta cell dysfunction associated to diabetes development. These results supports a role of ER stress in diabetes pathophysiology and contribute to the understanding of DD as a multi-organ syndrome.

## Background

Endoplasmic reticulum (ER) stress is a condition where an insult disrupts ER homeostasis and leads to accumulation of misfolded and unfolded proteins, which may lead to cellular dysfunction and even apoptosis [1]. In recent years, ER stress has been implicated in the pathophysiology of various diseases including diabetes, where it is involved in beta cell dysfunction [2]. Yet, while there are plenty of animal studies, few if any, human studies have examined if primary ER stress conditions are associated with impaired glucose metabolism per se. Darier disease (DD) is a hereditary skin condition caused by mutations in the *ATP2A2* gene encoding the sarco-endoplasmic reticulum ATPase 2 (SERCA2) calcium pump, which causes calcium dyshomeostasis and ER stress. Herein we examined the glucose metabolism of a previously genetically defined cohort of DD patients [3].

## Methods and results

We included 25 patients with DD and 25 healthy volunteers matched by age, gender and body mass index (BMI). Age matching was done in ± 5-year intervals and BMI was matched according to four categories: < 18.5, 18.5–24.99, 25–29.99 and > 30. Inclusion criteria were phenotype-positive individuals with histopathology-verified DD or phenotype-positive individuals with family history of DD. Exclusion criteria were pregnancy, oral corticosteroids, recent acute illness (past 4 weeks), active substance abuse, or severe kidney or liver disease. All patients, but one, were previously tested for *ATP2A2* mutations [3]. Since acitretin has a half-life of approximately 50 h and is known to alter glucose tolerance, subjects on oral acitretin treatment implemented a 7-day washout period before the visit; a longer washout was not considered ethical. An OGTT (75 g glucose) was performed in the morning after an overnight fast and in addition to glucose hemoglobin A1c (HbA1c), c-peptide, insulin, proinsulin was measured. Definitions of prediabetes and diabetes were made according to WHO guidelines. One control with diagnosed diabetes was excluded from OGTT in order to avoid the side effects of discontinuing diabetes medications. The Homeostasis Model Assessment (HOMA) is a computer model for assessing beta cell function (%B) and insulin resistance (insulin sensitivity, %S) from basal (fasting) glucose and insulin or c-peptide concentrations as percentages of a normal reference population and was used to assess %B and %S. DD subjects showed normal fasting glucose, oral glucose tolerance, proinsulin: insulin ratio, c-peptide, and HOMA2-%S, while HOMA2-%B was significantly higher (Table 1). To assess the potential effects of oral acitretin treatment on glucose homeostasis due to the possibility of low drug levels remaining as well as mutations, DD patients were sub-grouped into acitretin treated vs. not acitretin and pathogenic vs. benign mutations; however, no significant differences were observed (Table 2).

**Table 1:** Baseline characteristics and glucose metabolism

Baseline characteristics	DD patients	Control patients	*p*-value
*n*	25	25	
Age (years)	52 ± 13 (27–78)	51 ± 13 (27–76)	0.80^#^
Male sex	10 (40)	10 (40)	1.000^†^
BMI (kg/m^2^)	28.2 ± 5.3 (18.9–42.3)	27.0 ± 5.0 (19.8–38.7)	0.45^#^
Weight (kg)	81.4 ± 17.8 (54–119)	78.8 ± 18.2 (49.3–117)	0.73^#^
Height (cm)	169.6 ± 9.9 (152–193)	170.3 ± 11.5 (48.5–193)	0.83^#^
Current smoker	5 (20)	2 (8)	0.417^†^
DM family history	13 (52)	11 (44)	0.778^†^
Acitretin treatment	14 (56)	0 (0)	< 0.001^†^*
Hypertension treatment	3 (12)	4 (16)	1.000^†^
Dyslipidemia treatment	3 (12)	2 (8)	1.000^†^
Glucose metabolism			
Fasting plasma glucose (mmol/L)	5.3 ± 0.4	5.6 ± 0.5	0.07^#^
2-h plasma glucose (mmol/L)	5.6 ± 1.7	5.7 ± 1.3	0.68^#^
HbA1c (mmol/mol)	36±4	36 ± 4	0.36^#^
Beta cell function			
Proinsulin/insulin (ratio)	0.85 ± 0.32	0.80 ± 0.49	0.19^#^
C-peptide (nmol/L)	0.8 ± 0.2	0.7 ± 0.2	0.24^#^
HOMA2-%S HOMA2-%B	79.6 ± 32.7 122.7 ± 27.1	94.9 ± 52.4 103.5 ± 22.1	0.53^#^ 0.01^#^*

Continuous variables were expressed as mean ± standard deviation (minimum–maximum). Categorical values were expressed as a number (%). Two DD patients were adopted and heredity was unknown. Due to hemolysis, insulin levels from three DD and one control patient were excluded from the analysis

n, number; DD, Darier disease; BMI, body mass index; DM, diabetes mellitus; HbA1c, hemoglobin A1c

^#^Mann–Whitney U Test, ^†^Fisher’s Exact Test, * significant difference after Benjamini–Hochberg correction for multiple comparisons (p ≤ 0.05)

**Table 2:** Metabolic parameters in Darier disease patients sub-grouped for acitretin treatment and mutation variant pathogenicity

	DD acitretin	DD no acitretin	*p*-value^#^
*n*	14	11	
Fasting plasma glucose (mmol/L)	5.3 ± 0.3	5.4 ± 0.4	0.12
2-h plasma glucose (mmol/L)	5.6 ± 2.2	5.6 ± 0.6	0.76
HbA1c (mmol/mol)	35 ± 3	37 ± 4	0.62
Proinsulin/insulin (ratio)	0.85 ± 0.28	0.94 ± 0.41	0.63
C-peptide (nmol/L)	0.9 ± 0.2	0.7±0.2	0.05*
HOMA2-%B	134.6 ± 29.2	107.6±14.1	< 0.01*

	Pathogenic mutation variant	Benign mutation variant	*p*-value^#^
*n*	15	9	
Fasting plasma glucose (mmol/L)	5.2 ± 0.3	5.5 ± 0.4	0.04*
2-h plasma glucose (mmol/L)	5.6 ± 2.1	5.8 ± 1.0	0.77
HbA1c (mmol/mol)	37 ± 4	34 ± 2	0.02*
Proinsulin/insulin (ratio)	0.87 ± 0.30	0.83 ± 0.35	0.62
C-peptide (nmol/L)	0.8 ± 0.2	0.8 ± 0.2	0.55
HOMA2-%B	128.1 ± 30.0	115.4 ± 21.0	0.40

Note that *ATP2A2* mutation variant pathogenicity was previously determined by in silico prediction programmes [3]. Continuous variables were expressed as mean ± standard deviation (minimum–maximum)

n, number; DD, Darier disease; HbA1c, hemoglobin A1c

^#^Mann–Whitney U Test, *insignificant differences after Benjamini–Hochberg correction for multiple comparisons (p ≥ 0.05)

## Discussion

SERCA2 heterozygous mice show impaired cytosolic Ca^2+^, impaired insulin secretion and susceptibility to diet-induced diabetes [4]. Contrary to expectations, DD patients showed increased HOMA2-%B values, indicative of increased basal insulin secretion. This may be considered a type of dysfunction as increased basal insulin secretion values are associated with a worse clinical and metabolic phenotype in adults and adolescents and predicts deterioration of glucose control over time and thus type 2 diabetes [5]. HOMA2-%B values are shown to increase between 3–4 years prior to type 2 diabetes diagnosis, after which they steadily decrease until diagnosis [6]. Moreover, these data are supported by basic studies that showed thapsigargin induced SERCA2 dysfunction increased insulin secretion in vitro [7]. Taken together, the data indicates that DD patients may run a higher risk of developing diabetes which is supported by a recent study showing association with type 1 diabetes [8]. It is currently not known whether DD patients carry other risk factors for the development of diabetes irrespective of *ATP2A2* mutation status. However, since some DD patients seem to lack *ATP2A2* mutations altogether, speculations could be made as to the existence of possible diabetes risk factors other than mutation status per se for DD patients, for example skin inflammation, as inflammatory skin conditions such as psoriasis is linked with type 2 diabetes [9]. This is also in accordance to our data showing no significant difference between the pathogenic and benign mutation variants among DD patients (Table 2). We find it unlikely that acitretin use by DD patients would cause beta cell dysfunction as retinoids are associated with improved glycemic control [10] and was even suggested as novel diabetes drugs [11].

## Conclusions

Taken together, our study contributes to the growing body of evidence indicating that DD is a syndrome affecting multiple organs and not only the skin. Diabetes is easy to screen for and it appears reasonable to bear in mind the potential risk of diabetes when assessing DD patients, although we do not fully understand why this may be the case. Future larger studies may reveal how DD is associated with diabetes.

## Data Availability

Not applicable.

## Abbreviations

ER: Endoplasmic reticulum
DD: Darier disease
SERCA2: Sarco-endoplasmic reticulum ATPase 2
BMI: Body mass index
HOMA: Homeostasis Model Assessment
